# Solvent-Induced Polymorphism of Iron(II) Spin Crossover Complexes

**DOI:** 10.3390/ma9070585

**Published:** 2016-07-19

**Authors:** Ivan Šalitroš, Olaf Fuhr, Mario Ruben

**Affiliations:** 1 Institut für Nanotechnologie, Karlsruher Institut für Technologie, Postfach 3640, Karlsruhe 76021, Germany; olaf.fuhr@kit.edu; 2 Faculty of Chemical and Food Technology, Institute of Inorganic Chemistry, Technology and Materials, Slovak University of Technology, Bratislava 812 37, Slovakia; 3 Karlsruhe Nano Micro Facility (KNMF), Karlsruher Institut für Technologie, Postfach 3640, Karlsruhe 76021, Germany; 4 Institute de Physique et Chimie de Matériaux de Strasbourg (IPCMS), CNRS-Université de Strasbourg, 23, rue du Loess BP 43, F-67034 Strasbourg cedex 2, France

**Keywords:** spin crossover, iron(II) complexes, molecular magnetism, polymorphism

## Abstract

Two new mononuclear iron(II) compounds (**1**) and (**2**) of the general formula [Fe(**L**)_2_](BF_4_)_2_·nCH_3_CN (**L** = 4-(2-bromoethyn-1-yl)-2,6-bis(pyrazol-1-yl)pyridine, *n* = 1 for (**1**) and *n* = 2 for compound (**2**)), were synthesized. The room temperature crystallization afforded concomitant formation of two different solvent analogues: compound (**1**) exhibiting triclinic P-1 and compound (**2**) monoclinic C2/c symmetry. Single-crystal X-ray studies confirmed the presence of the LS (low-spin) state for both compounds at 180 K and of the HS (high-spin) state for compound (**2**) at 293 K, in full agreement with the magnetic investigations for both solvent polymorphs. Compound (**1**) exhibits spin transition above 293 K followed by subsequent solvent liberation, while the spin transition of (**2**) takes already place at 237 K. After complete solvent removal from the crystal lattice, compound (**1d**) (the desolvated polymorph derived from (**1**)) exhibits spin transition centered at 342 K accompanied by a thermal hysteresis loop, while the analogous compound (**2d**) (the desolvated derivate of compound (**2**)) remains blocked in the HS state over all the investigated temperature range.

## 1. Introduction

Spin crossover (SCO) can be considered as a unimolecular transformation upon which switching entities (e.g., molecular complexes or coordination polymers) are reversibly transformed between the low-spin (LS) and high-spin (HS) state. The transition of spin states is an entropy-driven process, which can be triggered by external parameters such as temperature [1], pressure [2], magnetic [3] or electric field [4,5], and by irradiation with the visible/near—infrared [6] or even X-ray [7] range. Up to now, the occurrence of the SCO phenomenon was observed for central atoms in coordination compounds of **3d**^4^–**3d**^7^ electronic configuration, whereby the spherical symmetry of *d*-orbitals was broken into the *e_g_* and *t*_2*g*_ orbital subsets by the tetragonal bipyramidal environment of the N_6_ coordination environment. From the thermodynamic point of view, the SCO can be described as a case of electronic bistability, which is associated with the existence of two minima of the Gibbs energy [8]. The LS state minimum holds the occupation of electrons according to the *Aufbau* principle, whereas the HS state minimum possesses an electronic configuration according to the Hund’s rule of maximal multiplicity. The separation between the reference states is proportional to the ligand field strength of 10 Dq. When the energy difference between the HS and LS states is of the order of *k*_B_*T* (where *T* is the temperature and *k*_B_ is the Boltzmann constant), switching between spin states on a molecular level can be stimulated by the change of temperature.

However, on the macroscopic scale, partially due to the extremely small energy differences, the ST is a highly subtle phenomenon, which can be easily affected by miscellaneous chemical [9] and/or structural [10,11] factors. The investigation of the environmental influences allows for tuning of the SCO parameters by, e.g., controlling the presence of solvent molecules in the crystal lattice [12]. In such way, the properties of SCO materials, requested to fulfill technological criteria such as abruptness, hysteresis and room-temperature transition [13] can be deliberately tailored. The technological advanced family of iron(II)-triazole [14] complexes and iron(II)-pyrazine-tetracyanometalate [15] networks are positive examples for such a molecular engineering approach. Beyond SCO coordination polymers, discrete molecular SCO species incorporating imidazole- [16], pyrazole- [17] or pyridine-type [18,19,20] ligands have been shown to exhibit room temperature transitions with moderate thermal hysteresis widths [21]. Within the last decade, the iron(II) complexes with tridentate bis(pyrazole)pyridine [22,23,24,25,26,27,28,29,30,31,32,33] ligands have drawn increasing of attention to the field of SCO complexes. It has been shown that the modification of the ligand skeleton by substituents on the pyridine [23,24,25,26] or pyrazole [27] moiety leads to a fine-tuning of SCO properties. This is in fact a powerful tool for the synthesis of room temperature SCO compounds with anchoring functional substituents, which are tailor-made for the fabrication of hybrid materials, i.e., SCO surfaces [28] or SCO nanoparticles [29]. With respect to this, also halogen substituents might significantly support the cooperativity effect via non-covalent interactions [29] as well as increase the ligand field strength and elevate the T_1/2_ temperature [27]. Another chemical factor affecting the SCO parameters is presence of the hosts, i.e., solvent molecules in the crystal lattice. The solvation/desolvation processes can actively change the transition behavior [31,32,33]. In this sense, the detailed study and understanding of the substituent effect and solvent-driven spin and phase transitions is one of the pivotal challenges for the SCO investigation.

Herein, we report on the synthesis, structural and magnetic investigation of two iron(II) SCO complexes (**1**) and (**2**), both containing the tridentate bis(pyrazol-1-yl)pyridine type of ligands. The coordination compound of the general formula [Fe(**L**)_2_](BF_4_)_2_ (**L** = 4-(2-bromoethyn-1-yl)-2,6-bis(pyrazol-1-yl)pyridine) exists in two different solvated and structural forms which differ in the number of acetonitrile molecules. Magnetic studies reveal that SCO is present in both solvent analogues (**1**) and (**2**). In situ solvent liberation within the magnetic measurements results in two different polymorphs of solvent-free compound [Fe(**L**)_2_](BF_4_)_2_ and magnetic investigation allowed us to recognize that compound (**1d**) (the desolvated polymorph derived from (**1**)) shows SCO centered at *T*_1/2_ = 342 K and compound (**2d**) (the desolvated polymorph derived from (**2**)) persists in the HS state over the whole investigated temperature range.

## 2. Results and Discussion

### 2.1. Synthesis, Crystallization and Thermogravimetry Analysis

The tridentate ligand **L**, 4-(2-bromoethyn-1-yl)-2,6-bis(pyrazol-1-yl)pyridine, was obtained as a white powder in almost quantitative yield via reaction of 4-ethynyl-2,6-bis(pyrazol-1-yl)pyridine [24,25,26] with N-bromosuccinimide, catalyzed by AgNO_3_ in acetone solvent. In the next step, ligand **L** was coordinated by the Fe(BF_4_)_2_·6H_2_O salt. The crystallization from the acetonitrile solution yielded two different compounds: (**1**) with the general formula [Fe(**L**)_2_](BF_4_)_2_·CH_3_CN and (**2**) with formula [Fe(**L**)_2_](BF_4_)_2_·2CH_3_CN. Due to the small size and similarity of the single crystals, separation of the two different solvent crystals was not successful.

**Figure 1:**
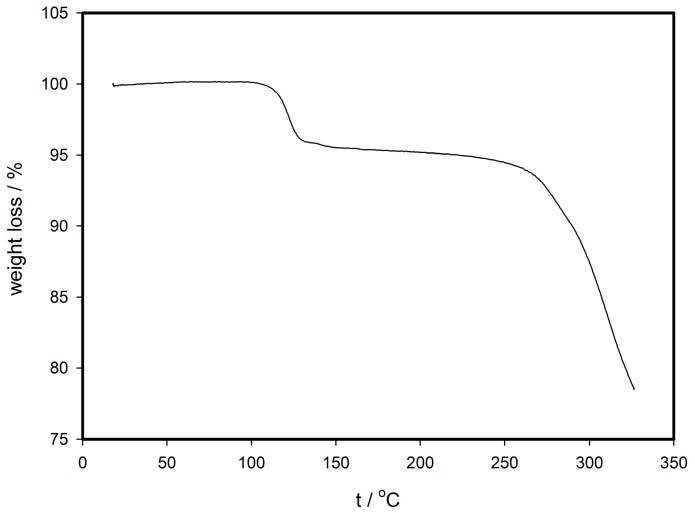
Thermogram of mixture sample of compounds (**1**) and (**2**).

Thermogravimetry analysis of the unknown mixture of (**1**) and (**2**) was performed at ambient pressure. The first decrease of about 5% of mass around 100 °C (Figure 1) is attributed to the release of acetonitrile molecules from the crystal lattice. Solvent-free compounds (**1d**) and (**2d**), derived from their solvated analogues, are remarkably stable up to 200 °C, and begin to decompose above this temperature. The fact that respective acetonitrile solvent molecules did not escape at their boiling point during the thermogravimetric measurements indicates a significant interaction of these species with the complex molecule through supramolecular bonds.

### 2.2. Structural Investigation

**Figure 2:**
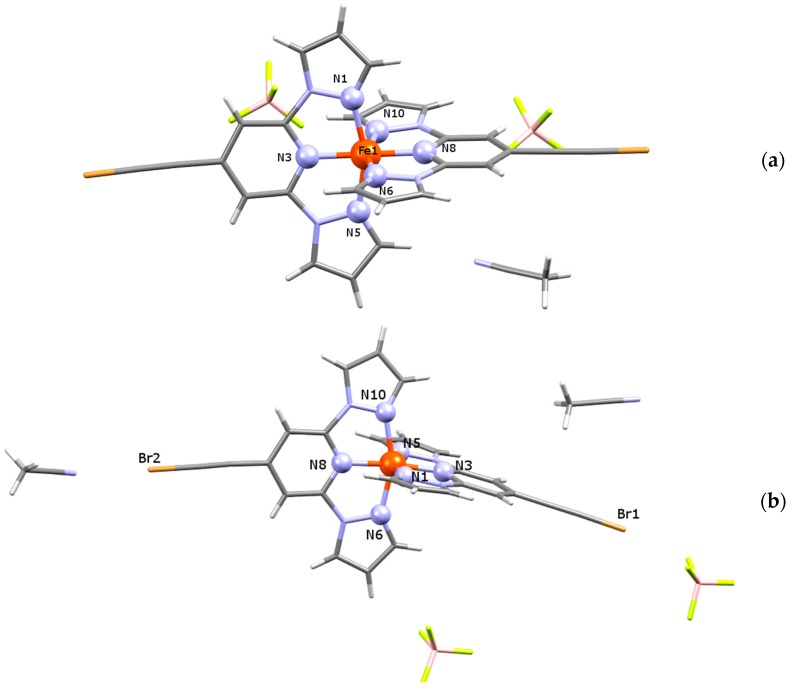
Asymmetric unit of solvated analogues (**a**) (**1**) and (**b**) (**2**).

At 180 K, the single-crystal X-ray diffraction study of (**1**) (Figure 2a) reveals a triclinic space group *P*-1 exhibiting a unit cell volume of V = 1713.9(6) Å^3^ and lattice parameters of *a* = 11.324(2) Å, *b* = 11.742(2) Å, *c* = 14.187(3) Å, α = 83.29(3)°, β = 84.40(3)°, γ = 66.38(3)° (Table 1). The asymmetric unit of (**1**) contains one complex [Fe(**L**)_2_]^2+^, two corresponding BF_4_^−^ counter anions and one CH_3_CN solvent molecule. Two molecular species of formula [Fe(**L**)_2_](BF_4_)_2_·CH_3_CN are present in the unit cell. At the temperature of the measurement, the Fe-N distances are in the range 1.887(3)–1.973(3) Å, indicating the iron(II) LS state (Table 2).

In order to investigate structural changes upon SCO (vide infra), the single-crystal X-ray structure of compound (**2**) was determined at 180 K (LS state) and at 293 K (HS state) using the same single crystal (Figure 2b). At both temperatures, the diffraction study elucidates a monoclinic system with *C*2/*c* symmetry, where the asymmetric unit contains one cationic complex [Fe(**L**)_2_]^2+^, two disordered BF_4_^−^ counter anions and two acetonitrile molecules. Unit cell parameters and other selected structural information are listed in Table 1. Coming from the LS to HS state, the unit cell volume increases about 4%, lattice parameters of *a* increase 1.5% and those of *b* increase 2.2%, respectively. The low temperature bond distances of a coordination polyhedron vary in the range 1.900(3)–1.998(3) Å and, likewise for (**1**), they indicate the LS state of the central atom. At 293 K, Fe-N lengths acquire values typical for the HS state of the central atom (2.098(3)–2.173(3) Å, Table 2) and in comparison to the LS structure, the most significant increase of distances is observed for the axial Fe1-N3 and Fe1-N8 bonds (≈10.4%), which are placed along the *b* direction. The equatorial Fe1-N6 and Fe1-N10 bonds situated along the *ab* plane show a variation between LS and HS distances of about 9.7% and 8.1%, and the third bond-couple Fe1-N1 and Fe1-N5 placed along the *bc* plane increases about 8.2% and 8.6%, respectively.

**Figure 3:**
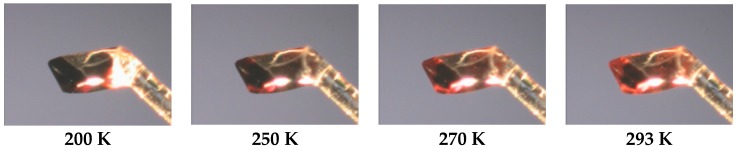
(Color on-line) Representation of the thermochromism of compound **2**.

The shape of coordination polyhedra of all three structures is a strongly deformed tetragonal bipyramid, whereby its plasticity can be expressed by the Σ parameter [34,35]. The LS Σ values 85.6° for (**1**) and 89.0° for (**2**), respectively, are significantly lower than that for the room temperature structure of (**2**) (Σ = 146.0°). Also, so-called bite angles (N_pz_-Fe-N_py_, Table 2) are good “indicators” for the change of the spin state in the family of bis(pyrazol-1-yl)pyridine-iron(II) compounds [9,10,11]. So the LS structures of (**1**) and (**2**) indicate values of 80.1° and 80.0°, respectively, while the HS value of compound (**2**) (at 293 K) with 74.1° is significantly smaller. The Jahn-Teller distortion of HS iron(II) molecules can be expressed also by the angle N_py_-Fe-N_py_, which is straight for (**1**) and (**2**) at 180 K but significantly bent at room temperature for (**2**) (Table 2). The detailed look into the monoclinic structure of (**2**) reveals short contacts between the endstanding bromine atoms of the **L** ligand with the acetonitrile nitrogen atoms on the one side of the molecule (2.9998(5) Å at 293 K, 2.9375(44) Å at 180 K), and with the BF_4_^−^ counter anion fluorine atoms (2.8342(61) Å at 293 K, 2.8375(6) Å at 180 K) on the other side of the molecule (Figure 2b). At 180 K, both structures show weak interactions between the ligand atoms and BF_4_^−^ counter anions. Those weak hydrogen binding and F···H-C contacts are shorter than sum of the van der Waals radii of F and H (2.55 Å) [36] and vary in the range of 3.1251(37)–3.3650(41) Å (with corresponding angles from 121.2(2)° to 170.4(2)°) for (**1**), and 3.1241(77)–3.3080(76) Å (with corresponding angles from 126.9(3)° to 162.4(2)°) for (**2**), respectively (see Supplementary Materials S1). For compound (**2**), neither the SCO nor the increases of temperature have a significant impact on the prolongation of the non-covalent interactions. Furthermore, the thermochromism related to SCO processes was investigated for single crystals of (**2**), whereby the initial dark orange changed gradually to light orange without losing its crystallinity (Figure 3).

### 2.3. Magnetic Properties of Compounds (**1**) and (**2**)

**Figure 4:**
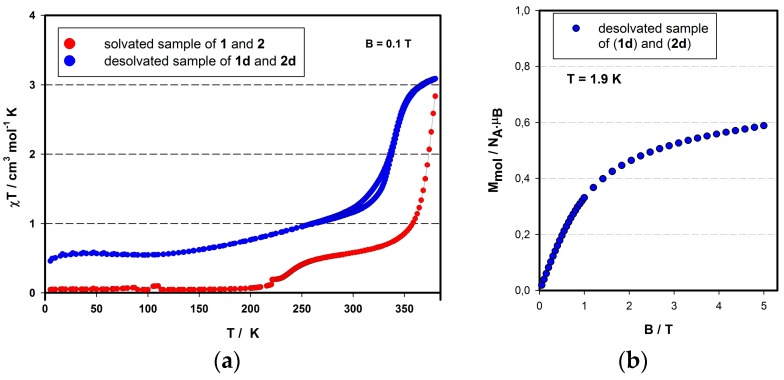
(**a**) Temperature-dependent magnetic properties of solvated (red circles) and desolvated (blue circles) mixture of compounds (**1**) [Fe(**L**)_2_](BF_4_)_2_·CH_3_CN and (**2**) [Fe(**L**)_2_](BF_4_)_2_·2CH_3_CN; (**b**) Field-dependent mole magnetization of desolvated sample at 1.9 K (mixture of (**1d**) and (**2d**)).

The high similarity of the crystals of (**1**) and (**2**) impeded a clean separation in order to separately investigate their magnetic properties. Therefore, we decided to study a sample of an unknown ratio of both compounds (**1**) ([Fe(**L**)_2_](BF_4_)_2_·CH_3_CN) and (**2**) ([Fe(**L**)_2_](BF_4_)_2_·2CH_3_CN), drawing conclusions inductively on the individual magnetic properties from the observed overall magnetic and crystallographic properties. According to the results from X-ray diffraction studies, both compounds are in LS state at 180 K, which is in good agreement with the variable temperature magnetic investigation of the (**1**)/(**2**) mixture (Figure 4a, red circles). Below 200 K, the product function of the measured sample acquires positive values close to zero, which can be explained by the small presence of paramagnetic impurities. Above 200 K, however, the sample exhibits an increase of the product function and reaches a plateau at room temperature. Keeping in mind that the room temperature bond distances of (**2**) indicate the HS state (vide supra), it can be concluded that the first spin crossover placed around 237 K belongs to compound (**2**). The *χT* product reaches 0.51 cm^3^·K·mol^−1^ at 290 K, which corresponds to a ratio of 17% of HS iron(II). Upon further warming above room temperature, the second spin crossover takes place. However, it is accompanied with the liberation of acetonitrile lattice solvent molecules. At 380 K, the *χT* function reaches 2.9 cm^3^·K·mol^−1^ which is very close to the expected value for the HS state of Fe(II); however, the SCO curve is not saturated yet. The loss of solvent molecules from crystal structures of (**1**) and (**2**) has created two polymorphic forms (**1d**) and (**2d**) of compound [Fe(**L**)_2_](BF_4_)_2_. The magnetic data of the desolvated sample (Figure 4a; blue circles) indicate a gradual spin crossover centered around 342 K. Moreover, the SCO is accompanied by a hysteresis loop, which is constant during all three heating cycles (see Supplementary Materials S2). Below 293 K, the SCO reaches the LS plateau at 0.5 cm^3^·mol^−1^·K (at 100 K) which corresponds to 17% of the HS mole fraction. The field dependency of molar magnetization of the desolvated mixture was carried out at 1.9 K (Figure 4b), whereby the saturation of *M*_mol_ is achieved around 0.6 μ_B_ at 5 T which deviates around 15% from the expected value for the HS mononuclear iron(II) compounds (S = 2).

On the grounds of the obtained results—(i) 17% of HS iron(II) observed at 293 K in the pristine sample; (ii) 17% of remnant HS fraction observed after desolvation; and (iii) 15% of HS iron(II) found according to the *M*_B_ vs. *B* measurement in the desolvated (**1d**)/(**2d**) sample—it can be concluded that the remnant HS molar fraction of the desolvated sample can exclusively come from the desolvated (**2d**) polymorph, while the above-room-temperature SCO behavior is due to the (**1d**) polymorph. In other words, it can be noticed that compound (**1**) [Fe(**L**)_2_](BF_4_)_2_·CH_3_CN possesses LS diamagnetic behavior up to room temperature, but releases acetonitrile solvent molecules above 300 K, subsequently leading to the SCO effect accompanied by a thermal hysteresis loop. On the other hand, compound (**2**) [Fe(**L**)_2_](BF_4_)_2_·2CH_3_CN exhibits a SCO centered at 237 K and looses acetonitrile solvent molecules at elevated temperatures to subsequently stay blocked in the HS.

## 3. Materials and Methods

### 3.1. General

Purchased chemicals (*N*-Bromosuccinimide, Fe(BF_4_)_2_·6H_2_O, AgNO_3_) were used as received. The compound 4-ethynyl-2,6-bis(pyrazol-1-yl)pyridine was prepared as described previously [25,26]. Acetone, hexane, ethyl acetate, acetonitrile and diethyl ether solvents were used without any further purification. Elemental analyses of carbon, hydrogen, and nitrogen were carried out by Vario Micro Cube. FT IR spectra were measured in KBr pellets (Magna FTIR 750, Nicolet, INT KIT, Karlsruhe, Germany in the 4000–400 cm^−1^ region. ^1^H and ^13^C NMR spectra were recorded in a Bruker DPX 300 spectrometer INT KIT, Karlsruhe, Germany) with solvent proton and carbon atoms as an internal standard. Matrix-assisted laser desorption/ionization time of flight (MALDI-TOF, INT KIT, Karlsruhe, Germany) mass spectrometric analytical data were acquired on a Voyager-DE PRO Bio spectrometry workstation (INT KIT, Karlsruhe, Germany). Electro-spray ionization time of flight (ESI-TOF) mass spectrometric analytical data were acquired on a microOTOF-Q II Bruker (INT KIT, Karlsruhe, Germany). Thermogravimetric analysis (TG) was performed in a He flow at a heating rate of 2.5 K·min^−1^ in a Netzsch STA 409 C analyzer (INT KIT, Karlsruhe, Germany).

### 3.2. Synthesis

The compound 4-ethynyl-2,6-bis(pyrazol-1-yl)pyridine was prepared according a reported procedure [26,27] via Sonoghashira coupling reaction of 4-iodo-2,6-bis(pyrazol-1yl)pyridine [24,25] with trimethylsilylacethylene in the first synthetic step and following deprotection of trimethylsilyl protecting group with methanol and sodium carbonate in the second step. The ligand preparation resulted in 79% yield as a white powder.

4-(2-bromoethyn-1-yl)-2,6-bis(pyrazol-1-yl)pyridine (**L**): 1 g (4.25 mmol) of 4-ethynyl-2,6-bis(pyrazol-1-yl)pyridine was dissolved in 100 cm^3^ of acetone, then 0.83 g (8.5 mmol) of N-bromosuccinimide and 60 mg (0.35 mmol) of AgNO_3_ were added. The resulted reaction mixture was stirred for 10 h under a N_2_ atmosphere at room temperature. The solvent was removed under vacuum using rotary evaporator and the resulting product was filtered on aluminium oxide with n-hexane/ethyl acetate (10:1; R_f_ = 0.64) as eluent. Due to the light sensibility of this compound, all manipulations dealing with **L** ligand were carried out in dark. ^1^H NMR (300 MHz, CDCl_3_, 25 °C, δ/ppm): 6.49 (td, 2H, pyrazole), 7.75 (dd, 2H, pyrazole), 7.87 (s, 2H, pyridine), 8.53 (d, 2H, pyrazole). ^13^C NMR (75 MHz, CDCl_3_, 25 °C, δ/ppm): 107.42, 112.19, 127.41, 136.28, 143.00, 150.56. MALDI-TOF MS: experiment: *m*/*z* (relative intensity of isotopic distribution) = 314.14 (100%), 315.13 (18%), 316.13 (95%); 317.10 (15%) simulation: *m*/*z* (relative intensity of isotopic distribution) = 313.00 (100%), 314.00 (17%), 316.13 (97%); 317.10 (15%). Elemental analysis for C_13_H_8_N_5_Br (314.14): Found (Calc.): C 50.00 (49.70)%; H 2.45(2.57)%; N 21.92 (22.29)%; yield 1.33 g (99%).

Synthesis of compounds [Fe(L)_2_](BF_4_)_2_·nCH_3_CN (*n* = 1, (**1**); *n* = 2 (**2**)): An acetonitrile solution of **L** (0.1 g, 0.32 mmol, 50 cm^3^) was deoxygenated under the N_2_ flux, warmed-up to 60 °C and then a stoichiometric amount of Fe(BF_4_)_2_·6H_2_O (0.054 g, 0.16 mmol) was added. The complexation of ligand **L** took place under color change to red-orange. The resulting reaction mixture was stirred at initial conditions (60 °C, N_2_ atmosphere) for 3 h, cooled down to room temperature and filtered. Dark orange block-shaped crystals were grown from diffusing the diethyl ether into an acetonitrile solution of the complex under N_2_ at room temperature. The diffraction study of single crystals revealed concomitant formation of two different solvent analogues -compound (**1**) with the formula [Fe(**L**)_2_](BF_4_)_2_·CH_3_CN and compound (**2**) of formula [Fe(**L**)_2_](BF_4_)_2_·2CH_3_CN. Unfortunately, the small size and similarity of single crystals did not allow for clean separation of compounds (**1**) and (**2**). As consequence, the mixture of the unknown ratio of those two solvent polymorphs was used for further analytical and physical property studies. Calc. for C_28_H_19_B_2_Br_2_F_8_FeN_11_ (898.75 g/mol) Found (Calc.): C 37.61 (37.42)%; H 2.41 (2.13)%; N 17.12 (17.14)%. ESI-TOF MS: [Fe(**L**)_2_]^2+^ (FeC_26_H_16_Br_2_N_10_)at *m*/*z* = 341.9681 (calc. *m*/*z* =341.9624); [Fe(**L**)_2_(BF_4_)]^+^ (FeC_26_H_16_Br_2_N_10_BF_4_))at *m*/*z* = 770.9441 (calc. *m*/*z* = 770.9286); [Fe(**L**)_2_(BF_4_)_2_]Li^+^ (FeC_26_H_16_Br_2_N_10_B_2_F_8_Li) at *m*/*z* = 864.8824 (calc. *m*/*z* = 864.9481). ^1^H NMR (300 MHz, CH_3_CN, 25 °C, *δ*/ppm): 56.82 (s, pyrazole), 52.01 (s, pyridine), 34.45 (pyrazole), 31.92 (s, pyrazole). FT IR (KBr; $\bar{v}$ /cm^−1^): 3155 (ms, C–H_ar_), 3140 (ms, C–H_ar_); 3133 (ms, C–H_ar_), 3115 (ms, C–H_ar_); 2205 (ms, C≡C). Yield 0.10 g (71%).

### 3.3. Magnetic Susceptibility Measurement

All herein reported magnetic measurements were performed on a SQUID magnetometer (Quantum Design, model MPMS-XL-5, INT KIT, Karlsruhe, Germany). In all cases, the temperature dependence of the product function was recorded at *B* = 0.1 T as an external magnetic field. The temperature sweeping rate was 1 K·min^−1^ and was the same for cooling and heating mode. Gelatine capsules were used as sample containers for the measurement in the temperature range 5–380 K. Desolvation of (**1**) and (**2**) to (**1d**) and (**2d**) was obtained in situ within the magnetic measurement set-up. After first heating, three continuous cooling/heating cycles were applied until the last two measurements were identical. Thereby, the sample was maintained in the MPMS magnetometer at 380 K for 20 min before every cooling/heating cycle. The very small diamagnetic contribution of the gelatine capsule and high-temperature sample holder had a negligible contribution to the overall magnetization. The diamagnetic corrections of the molar magnetic susceptibilities were applied using Pascal’s constants [9].

### 3.4. Single-Crystal Diffraction

Single-crystal X-ray diffraction data were collected on a STOE IPDS II diffractometer with graphite monochromated Mo Kα radiation (0.71073 Å). Structures were solved by direct methods (SHELX-97). Refinement was performed with anisotropic temperature factors for all non-hydrogen atoms (disordered atoms were refined isotropically) [37,38].

## 4. Conclusions

In conclusion, the results presented illustrate the effect of removal of auxiliary lattice solvent molecules as well as the influence of bromine substitution on the spin crossover. The solvent-induced polymorphism of the system [Fe(**L**)_2_](BF_4_)_2_·nCH_3_CN was studied and SCO behavior dependent on host solvent molecules was observed. The polymorphs containing slightly different amounts of solvent molecules in the crystal lattice differ strongly in their magnetic properties, mostly due to the formation of different crystal lattice symmetries. However, after solvent removal, the magnetic behaviors of the desolvated samples also differ strongly in their properties. On the other hand, the introduction of bromo substituent onto the periphery of 4-ethynyl-2,6-bis(pyrazol-1-yl)pyridine significantly strengthens the intermolecular interactions between the complex cation and BF_4_^−^ counter anions and/or solvent molecules. This type of interaction is missing in the case of “parental” iron(II) complexes with unsubstituted ligands [26,27], which is one of the reasons why related compounds exhibited such different spin crossover behavior. Our results highlight the importance of the control of the crystal lattice environment in ST materials, a problem to be taken into account in the design of molecular devices based on ST materials.

## Figures and Tables

**Table 1:** Crystal structure parameters for compounds (**1**) and (**2**).

	Compound (1) [Fe(L)_2_](BF_4_)_2_·CH_3_CN	Compound (2) [Fe(L)_2_](BF_4_)_2_·2CH_3_CN	
Formula	C_28_H_19_B_2_Br_2_F_8_FeN_11_	C_30_H_22_B_2_Br_2_F_8_FeN_12_	C_30_H_22_B_2_Br_2_F_8_FeN_12_
Formula weight/g·mol^−1^	898.83	939.89	939.89
Crystal color	Red	orange	orange
Temperature/K	180(2)	180(2)	293(2)
Wavelength/Å	0.71073	0.71073	0.71073
Crystal system	Triclinic	monoclinic	monoclinic
Space group	*P*-1	*C*2/*c*	*C*2/*c*
*a*/Å	11.324(2)	24.948(5)	25.311(5)
*b*/Å	11.742(2)	20.260(4)	20.702(4)
*c*/Å	14.187(3)	18.360(4)	18.395(4)
α/°	83.29(3)	90.00	90.00
β/°	84.40(3)	129.92(3)	129.81(3)
γ/°	66.38(3)	90.00	90.00
V/Å^3^	1713.9(6)	7117(3)	7404(3)
*Z*, *ρ*_calc_/g·cm^−3^	2, 1.742	8, 1.754	8, 1.686
μ (Mo-K_α_)/mm^−1^	2.855	2.755	2.648
*F*(000)	884	3712	3712
Crystal size/mm	0.35 × 0.33 × 0.16	0.41 × 0.32 × 0.24	0.41 × 0.32 × 0.24
θ range for the data collection/°	1.45 to 25.64	1.46 to 25.62	1.34 to 25.79
Final *R* indices [*I* > 2σ(I)] *	*R*_1_ = 0.0371 *wR*_2_ = 0.0929	*R*_1_ = 0.0375 *wR*_2_ = 0.0852	*R*_1_ = 0.0516 *wR*_2_ = 0.1314
*R* indices (all data) *	*R*_1_ = 0.0453 *wR*_2_ = 0.0964	*R*_1_ = 0.0525 *wR*_2_ = 0.0902	*R*_1_ = 0.0707 *wR*_2_ = 0.1417
GoF on *F*^2^	1.046	1.048	1.035
CCDC deposit number	846337	846338	846339

* $R_{1}=\sum^{\text{​}}\left(F_{0}-F_{c}\right)/\sum^{\text{​}}F_{0};\;\;wR_{2}=\sqrt{\frac{\sum^{\text{​}}\left[w\left({F_{0}}^{2}-{F_{c}}^{2}\right)\right]}{\sum^{\text{​}}[w{\left({F_{0}}^{2}\right)}^{2}]}}$.

**Table 2:** Fe-N bond distances (in Å) for (**1**) and (**2**).

(1) 180(2) K	(2) 180(2) K	(2) 293(2) K
Fe1-N1 = 1.973(2)	Fe1-N1 = 1.971(3)	Fe1-N1 = 2.133(4)
Fe1-N3 = 1.887(2)	Fe1-N3 = 1.906(3)	Fe1-N3 = 2.104(3)
Fe1-N5 = 1.972(2)	Fe1-N5 = 1.981(3)	Fe1-N5 = 2.151(4)
Fe1-N6 = 1.973(3)	Fe1-N6 =1.981(3)	Fe1-N6 =2.173(4)
Fe1-N8 = 1.895(2)	Fe1-N8 = 1.900(3)	Fe1-N8 = 2.098(3)
Fe1-N10 = 1.962(2) N_pz_-Fe-N_py_ * = 80.1 N3-Fe-Fe8 = 179.3	Fe1-N10 = 1.998(3) N_pz_-Fe-N_py_ = 79.9 N3-Fe-Fe8 = 173.0	Fe1-N10 = 2.160(4) N_pz_-Fe-N_py_ = 74.1 N3-Fe-Fe8 = 167.7

* Average values.

## Supplementary Materials

The following are available online at www.mdpi.com/1996-1944/9/7/585/s1.
