# Supplementary material for: Solvent-Induced Polymorphism of Iron(II) Spin Crossover Complexes
Source: Materials (Basel). 2016 Jul 19;9(7):585. doi: 10.3390/ma9070585 (PMC5456902; doi:10.3390/ma9070585)
Supplement: Supplementary file 1 [file materials-09-00585-s001.pdf]

Supplementary Materials: Solvent-Induced Polymorphism of Iron(II) Spin Crossover Complexes

Ivan Šalitroš, Olaf Fuhr and Mario Ruben

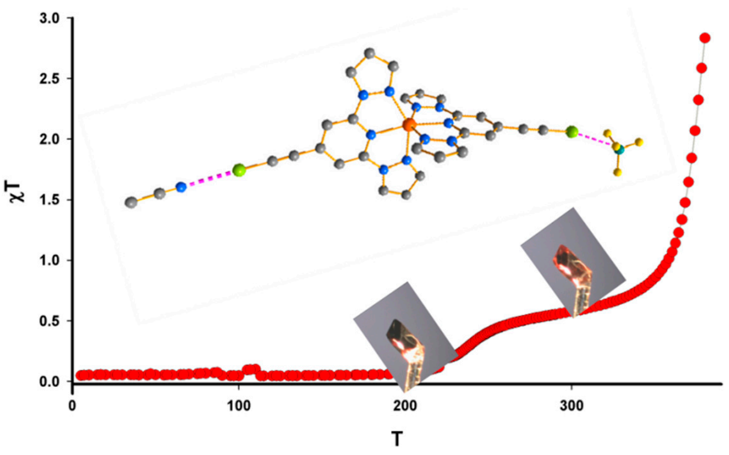

Figure S1. Crystallization of complex [Fe(**L**)$_2$](BF_4)$_2$ resulted in formation of two different solvent analogues of general formula [Fe(**L**)$_2$](BF_4)$_2$·nCH_3CN (**L** = 4-(2-bromoethyn-1-yl)-2,6-bis(pyrazol-1-yl) pyridine; *n* = 1 for compound (**1**); *n* = 2 for compound (**2**)). Crystal structures of both compounds exhibited low spin state at 180 K and compound (**2**) shoved high spin state at 293 K. Magnetic investigation confirmed low spin–high spin transition of compound (**2**) placed bellow the room temperature and revealed presence of spin transition centered at T_c = 342 K, accompanied with thermal hysteresis loop in the case of solvent free polymorph arisen from compound (**1**).

S. No.	Contents	Chapter
1	Hydrogen bonds and short contacts distances in crystal structures of (**1**) and (**2**)	S1
2	Solvent driven changes in magnetic behaviour of compounds **1** and **2**	S2

S1 Hydrogen Bonds and Short Contacts Distances in Crystal Structures of (1) and (2)

Table S1. Selected noncovalent contacts for compound (**1**) (at 180 K).

Bonds	F-C (in Å)	F-H (in Å)	Angle (F-C-H) (in deg)
F3C7	3.2654(46)	2.5034(25)	137.269(221)
F2C16	3.2526(35)	2.3739(21)	153.828(182)
F3C9	3.1686(37)	2.4384(21)	133.509(226)
F1C1	3.1467(37)	2.2679(21)	153.528(209)
F3C24	3.2840(36)	2.4869(21)	141.520(183)
F1C18	3.1697(36)	2.2372(20)	166.686(178)
F6C22	3.1251(37)	2.5258(24)	121.224(203)
F6C28	3.2451(78)	2.3028(27)	160.992(392)
F7C20	3.3650(41)	2.4247(29)	170.407(222)

Table S2. Selected noncovalent contacts for compound (**2**) (at 180 K).

Bonds	F-C (in Å)	F-H (in Å)	Angle (F-C-H) (in deg)
F1ABr1	2.8342(61)	N12Br2	2.9375(5)
F1BC1	3.2484(101)	ππ	133.696(542)
F2AC1	3.3054(72)	2.3931(542)	160.992(278)
F4AC30	3.2167(116)	2.3070(95)	154.236(336)
F7BC23	3.1916(93)	2.4691(73)	126.946(314)
F7BC7	3.159(118)	2.4139(100)	135.176(293)
F7AC7	3.2606(118)	2.4967(100)	137.459(293)
F7AC9	3.1241(77)	2.4212(68)	130.637(267)
F3BC22	3.2546(72)	2.3371(65)	162.452(257)
F3ac22	3.3080(76)	2.4232(70)	155.061(256)
F4ac30	3.2167(116)	2.3070(95)	154.236(336)
F8BC28	3.2629(124)	2.3683(98)	154.788(733)

S2 Solvent Driven Changes in Magnetic Behaviour of Compounds 1 and 2

Desolvatation of mixture sample of 1 and 2 compounds was carried out in situ within the magnetic measurements, by one heating and following three continuous cooling/heating cycles and maintaining the sample in the MPMS magnetometer at 380 K for 200 min before every cooling/heating cycle, until the last and next to last measurement cycle were identical. The thermal hysteresis loop of desolvated signal is permanent and survived all three cooling/heating cycles (Figure S2).

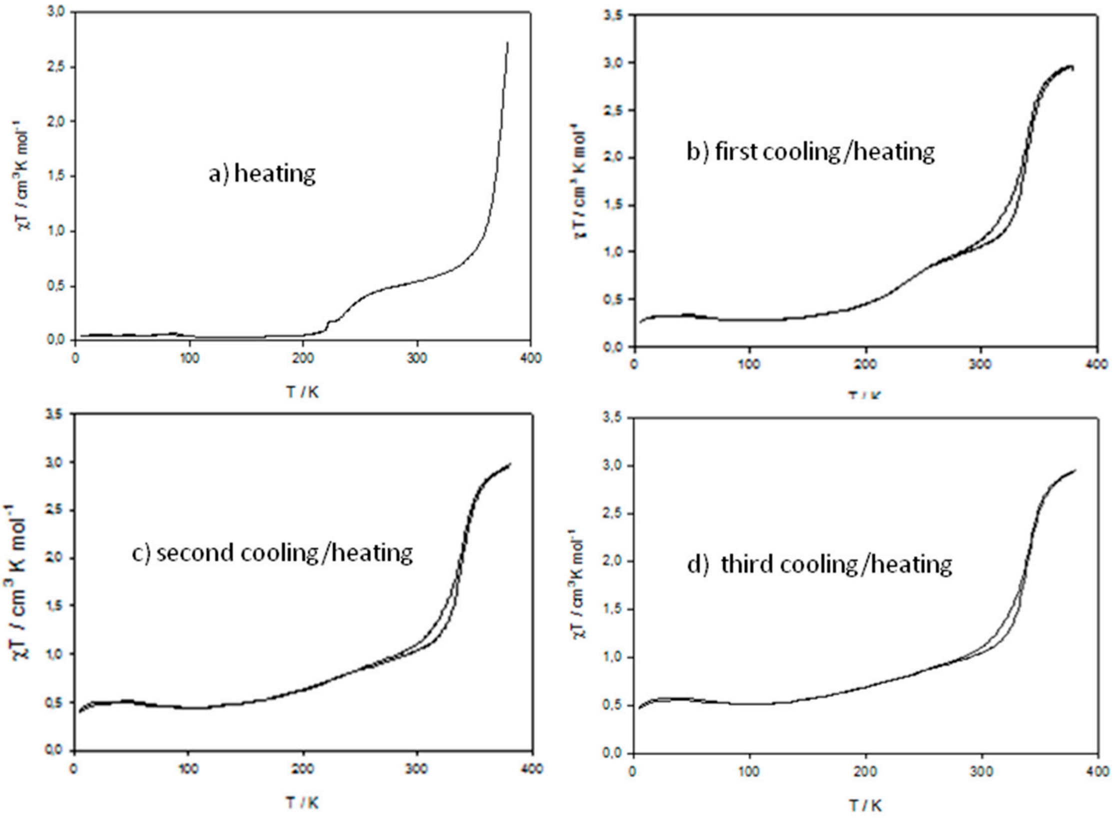

Figure S2. *Cont.*

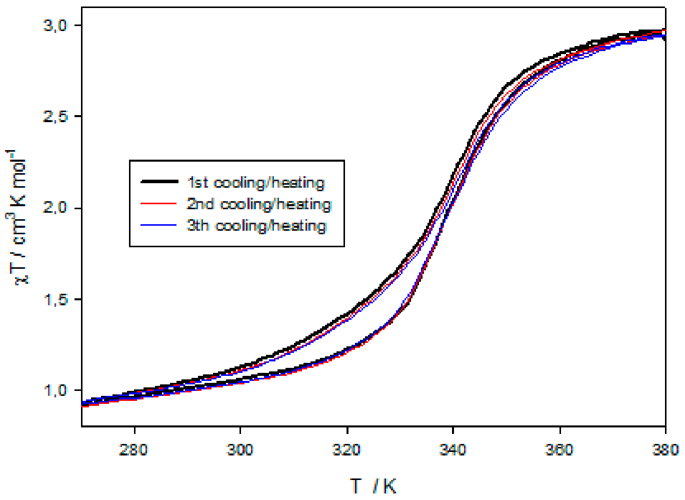

e)

Figure S2. (**a**–**d**) In situ desolvatation of compounds (**1**) and (**2**) within four cooling/heating cycles and (**e**) detailed look on the thermal hysteresis loop which survived the desolvatation process.
